# 16S rRNA Amplicon Sequencing of Sediment Bacterial Communities in an Oyster Farm in Rhode Island

**DOI:** 10.1128/MRA.01074-19

**Published:** 2019-10-17

**Authors:** Joshua T. E. Stevens, Robinson W. Fulweiler, Priyanka Roy Chowdhury

**Affiliations:** a Department of Biology, Keene State College, Keene, New Hampshire, USA; b Department of Earth & Environment, Boston University, Boston, Massachusetts, USA; c Department of Biology, Boston University, Boston, Massachusetts, USA; Georgia Institute of Technology

## Abstract

Little is known about the impact of oyster farming on sediment microbial communities. Here, we use 16S rRNA gene sequencing to identify bacterial communities in 24 sediment samples collected from an oyster farm in Ninigret Pond, RI. A total of 13,147 unique operational taxonomic units (OTUs) were assigned, with *Proteobacteria* being the dominant phyla across all samples.

## ANNOUNCEMENT

For over a decade, aquaculture has been a primary seafood-harvesting method, accounting for nearly half of the marine life consumed (1). As the demand for seafood has increased, aquaculture practices have become more common, especially in coastal regions (2). Oyster aquaculture is rapidly expanding along the coastlines of the United States. Oysters are ecological engineers that provide a range of ecosystem services, including reducing suspended sediment loads, filtering nutrients, and providing habitat for diverse marine biota (3). One area ripe for exploration is quantifying how oysters alter the adjacent sediment microbial community (4).

Here, we report 16S rRNA gene sequencing data of sediment microbial communities collected from a commercial oyster farm (0.016 km^2^) in a shallow coastal lagoon (Ninigret Pond) in southern Rhode Island (41.357°N, 71.6534°E). In June 2015, we collected sediment samples from 4 sites, located within the farm, each with different durations of oyster farming (0, 3, 5, and 7 years) (5). Using a 25-mm corer, we collected duplicate samples from the top 1 cm of sediments under oyster cages from 3 locations within each of the 4 sites. Sediment samples were placed on dry ice for transport back to the laboratory and stored at –80°C for future DNA extraction. DNA was isolated using a Qiagen DNeasy PowerSoil pro kit (catalog number 47014). Quality and quantity of DNA were measured using NanoDrop (NanoDrop Technologies, DE) and Qubit instruments (Thermo Fisher, Cambridge, MA) instruments, respectively. 16S rRNA amplicon sequencing was conducted at MR DNA (Shallowater, TX) on an Ion Torrent personal genome machine (PGM). Before sequencing, the 16S rRNA V4 variable region was amplified with primers 515F and 806R (6) using the HotStarTaq plus master mix kit (Qiagen, USA). Single-end sequences (1 × 150 bp) were processed using an MR DNA analysis pipeline that removed barcodes and primers, denoised sequences, and eliminated any sequences of <150 bp. Operational taxonomic units (OTUs) with 97% similarity cutoff were then clustered using USEARCH and classified using BLASTN against a database derived from Ribosomal Database Project II (RDP-II; http://rdp.cme.msu.edu) and NCBI (www.ncbi.nlm.nih.gov) after identification and removal of chimeras using a modified UCHIME algorithm (7). OTU count data were normalized to uniform read depth across samples. Shannon and Chao diversity indices were calculated using the *vegan* package in R (v. 3.5.3) (8).

**FIG 1:**
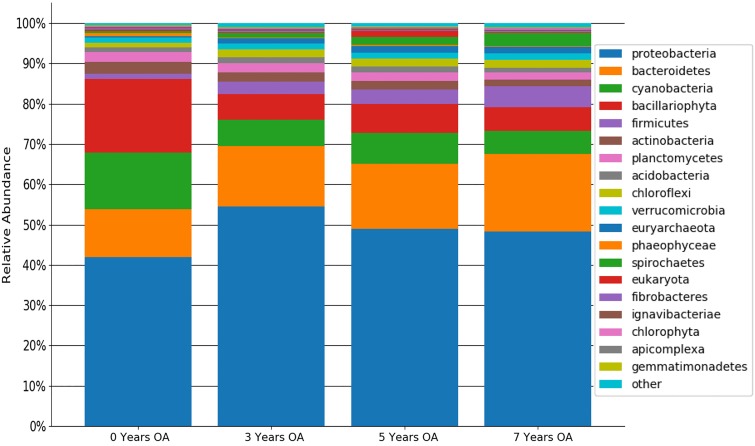
Phylum taxonomy based on relative OTU abundances in all 4 study sites. Each column represents control and 3, 5, and 7 years of oyster aquaculture (OA). Colored bars represent average phylum abundance in all 6 replicates normalized to per million annotated OTUs in that sample. The plot was created in Python (v. 2.7) using default parameters.

The number of reads/sample varied from 42,084 to 129,134 bp across the 24 samples, with GC contents ranging from 50% to 52%. Results showed 13,147 unique OTUs in all samples, with the least diverse sample possessing 3,990 OTUs and the most diverse containing 8,039 OTUs. The Shannon index indicated a slightly increased diversity in the sites with oyster aquaculture compared with that of the control (control, 6.247 to 6.617; 3 years, 6.622 to 7.246; 5 years, 6.797 to 7.218; 7 years, 6.476 to 7.245), while alpha diversity species richness (Chao) indicated a similar trend in the control compared with the farmed sites (control, 6,561.23 to 8,059.65; 3 years, 8,198.05 to 10,937.86; 5 years, 8,953.25 to 9,214.86; 7 years, 8,185.34 to 9,438.57). Bacterial OTUs were assigned to 53 different phyla, 432 different families, and 1,137 different genera. Relative abundances of common phyla are summarized in Fig. 1. Across all 4 sites, the dominant taxa were comprised of *Proteobacteria* (38% to 63%), *Bacteroidetes* (9.3% to 23%), *Cyanobacteria* (3.4% to 16%), and *Bacillariophyta* (3.2% to 24%). Overall, farm age did not affect phylum abundances (2-way analysis of variance [ANOVA], *P* > 0.99). Further comparisons of the relative abundances of individual taxa across different sites will help identify the short- and long-term impact of oyster farming on sediment bacterial communities that live in close association with the oysters.

### Data availability.

16S rRNA amplicon DNA sequences from this study have been uploaded to the DDBJ Sequence Read Archive (SRA) under the accession number PRJNA561593.
